# Supplementary material for: Dissecting diazirine photo-reaction mechanism for protein residue-specific cross-linking and distance mapping
Source: Nat Commun. 2024 Jul 18;15:6060. doi: 10.1038/s41467-024-50315-y (PMC11258254; doi:10.1038/s41467-024-50315-y)
Supplement: Supplementary file 1 — Supplementary Information [file 41467_2024_50315_MOESM1_ESM.pdf]

Supplementary Information for

Dissecting Diazirine Photo-reaction Mechanism for Protein Residue-Specific Cross-linking and Distance Mapping

Yida Jiang[1], Xinghe Zhang[1], Honggang Nie[1,2], Jianxiong Fan[1], Shuangshuang Di[1,2], Hui Fu[1,2], Xiu Zhang[1,2], Lijuan Wang[1,2], Chun Tang[1,3*]

[1]Beijing National Laboratory for Molecular Sciences, College of Chemistry and Molecular Engineering, Peking University, Beijing 100871, China. [2]Analytical Instrumentation Center, College of Chemistry and Molecular Engineering, Peking University, Beijing 100871, China. [3]Center for Quantitative Biology, PKU-Tsinghua Center for Life Sciences, Academy for Advanced Interdisciplinary Studies, Peking University, Beijing 100871, China

Email: Tang_Chun@pku.edu.cn

Supplementary Figures

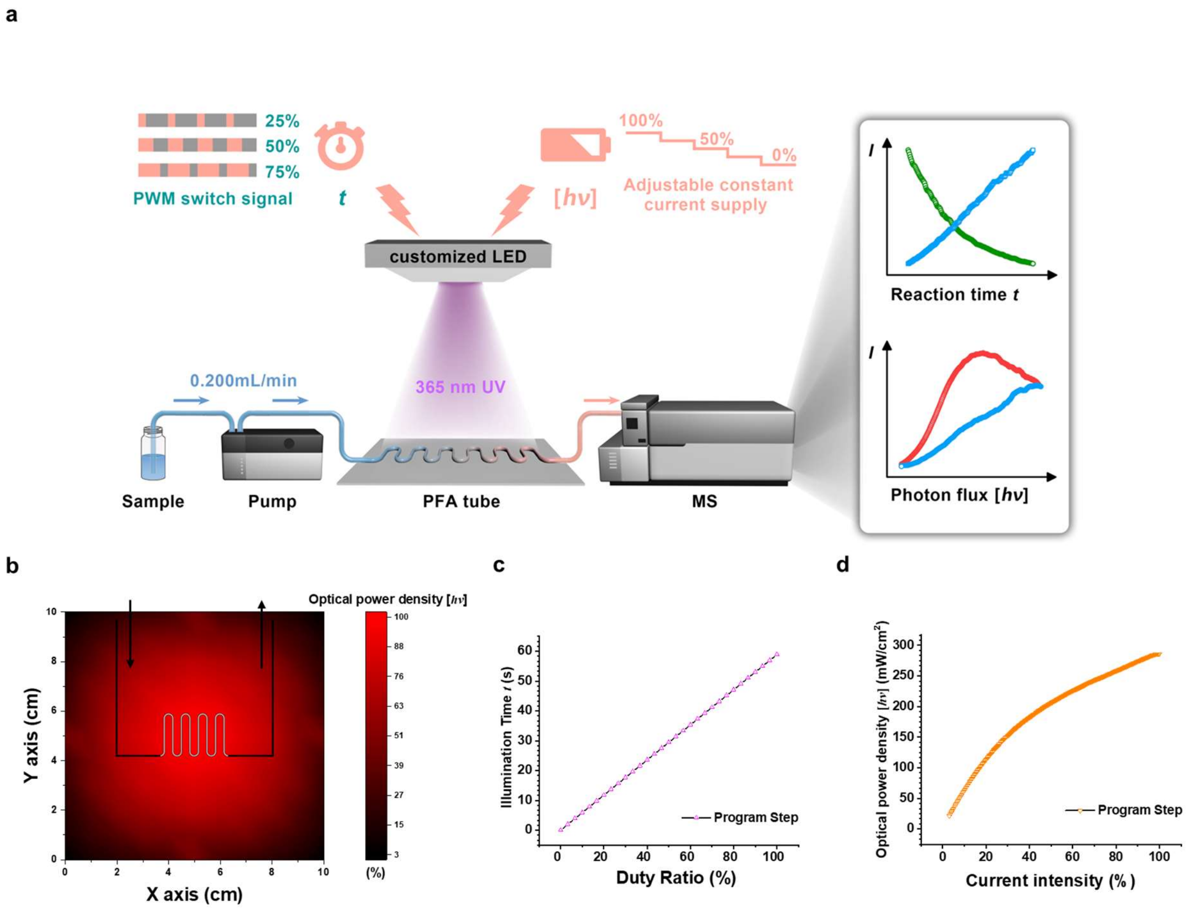

Supplementary Figure 1 | Setting up a power-modulated photo-reaction system with in-line MS monitoring. a, Reaction takes place in the PFA tube arranged zigzaggedly under a uniform light field, with the sample injected in a constant flow. Both irradiation time *t* and optical power density $[\boldsymbol{hv}]$ can be modulated to generate a large array of data points, allowing for the fitting of kinetic parameters (insets). Reaction mixture is subsequently injected to MS for real-time Multiple Reaction Monitoring (MRM) analysis. **b**, Calibration of the irradiation field over the PFA sample tube, illustrating homogeneity reached. **c**, Modulation of irradiation time *t* by using the scheme of pulse-width modulation (PWM), with 31 duty ratios programmed. **d**, Modulation of the optical power density $[\boldsymbol{hv}]$ with adjustable constant current supply; 256 current modes, corresponding $[\boldsymbol{hv}]$ from 0.1 to 289 mW/cm^2, can be programmed.

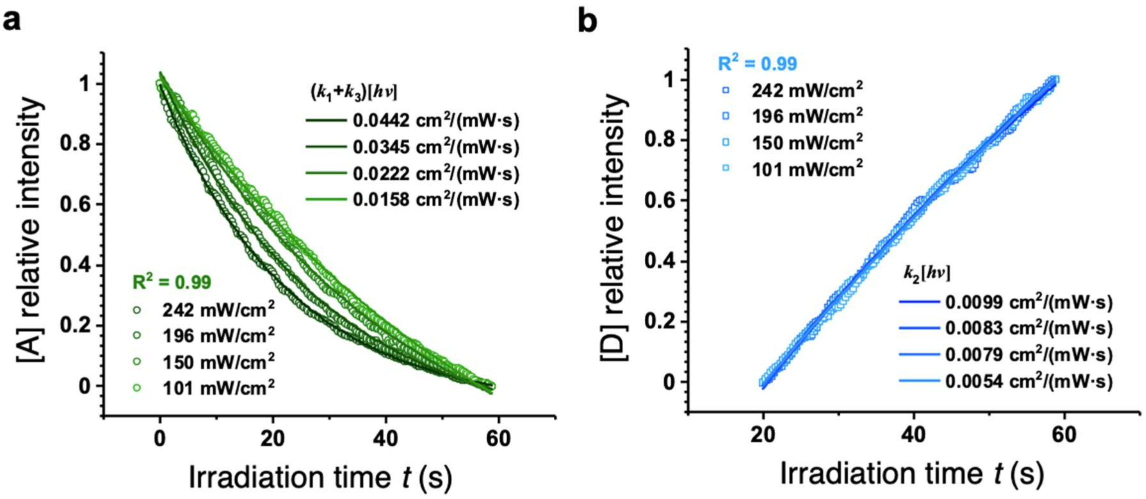

Supplementary Figure 2 | Fitting of the time-dependent relative concentration changes of [*A*] and [*D*]. a, Schematic diagram of single-exponential fitting of ***A*** to obtain $(k_1 + k_3)[hv]$. At a particular optical power density $[hv]$, [***A***] was fitted using equation of $I_A = [A]_0 exp(-(k_1 + k_3)[hv]t) + I_0$. **b**, Schematic diagram of fitting of [***D***] to obtain $k_2[hv]$, with irradiation time *t* > 20 s. Time-dependent [***D***] can be described with the equation $I_D = [A]_0 \left(1 - \frac{k_1}{k_1 - k_2 + k_3} exp(-k_2[hv]t) + \frac{k_2 - k_3}{k_1 - k_2 + k_3} exp(-(k_1 + k_3)[hv]t)\right) + I_0$. Four irradiation intensities are plotted, with details of the equations provided in the **Supplementary Note**.

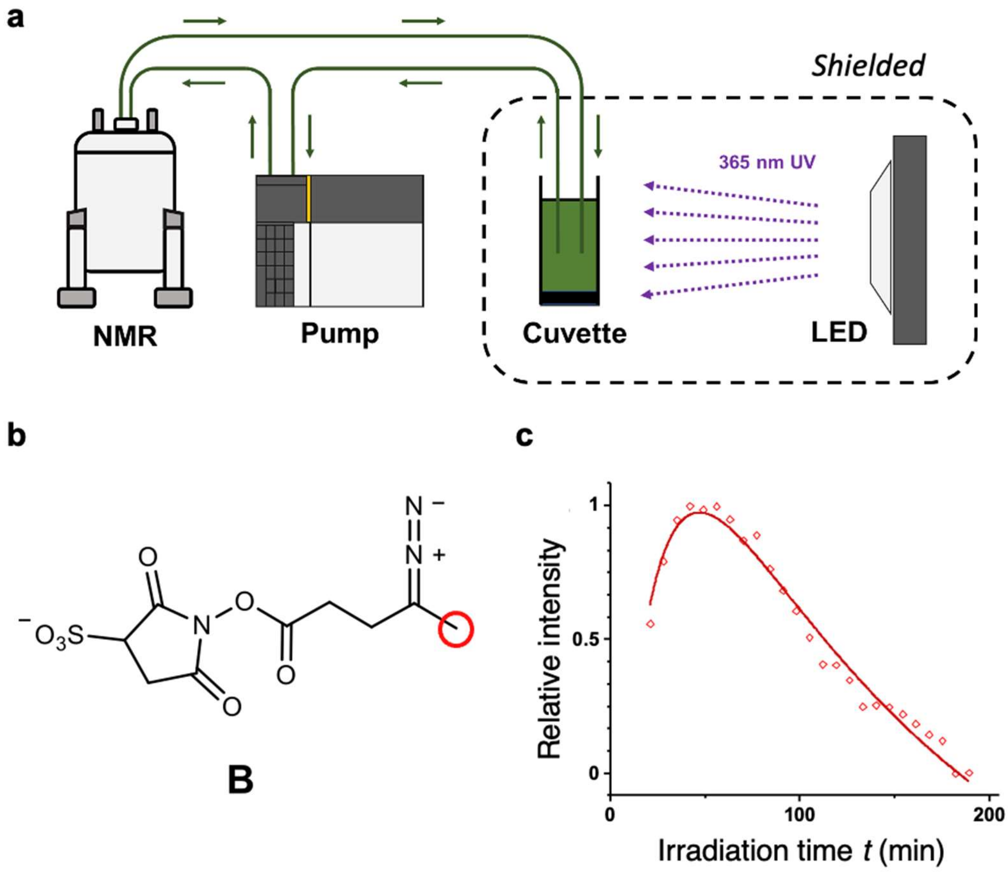

Supplementary Figure 3 | Real-time NMR monitoring of the diazo intermediate *B*. **a**, An in-line NMR spectroscopy setup, which allows for UV irradiation and NMR measurement of the product. **b-c**, The structure of diazo intermediate ***B***. The atom denoted, with a characteristic NMR peak at ~0.99 ppm, of which the intensity follows a curve that can be described with model **I** or **II**. The peak area (intensity) was integrated and normalized to the highest value.

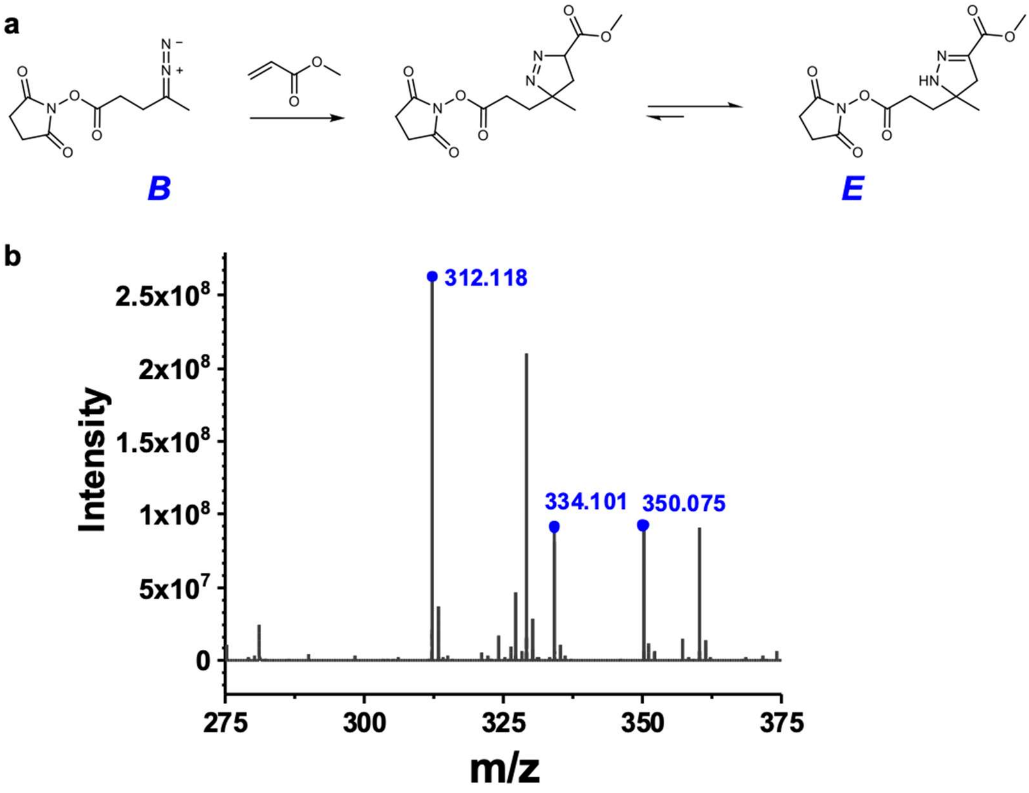

Supplementary Figure 4 | Capturing the diazo intermediate *B* with methyl methacrylate. a, The reaction between diazo intermediate ***B*** and methyl methacrylate, affording compound ***E***. **b**, ***E*** was confirmed in Fourier Transform Ion Cyclotron Resonance Mass Spectrometer (Solarix XR, Bruker) with the observed m/z of 312.118 ($[M+H]^+$), 334.101 ($[M+Na]^+$), and 350.075 ($[M+K]^+$). Expected, 311.112 Da.

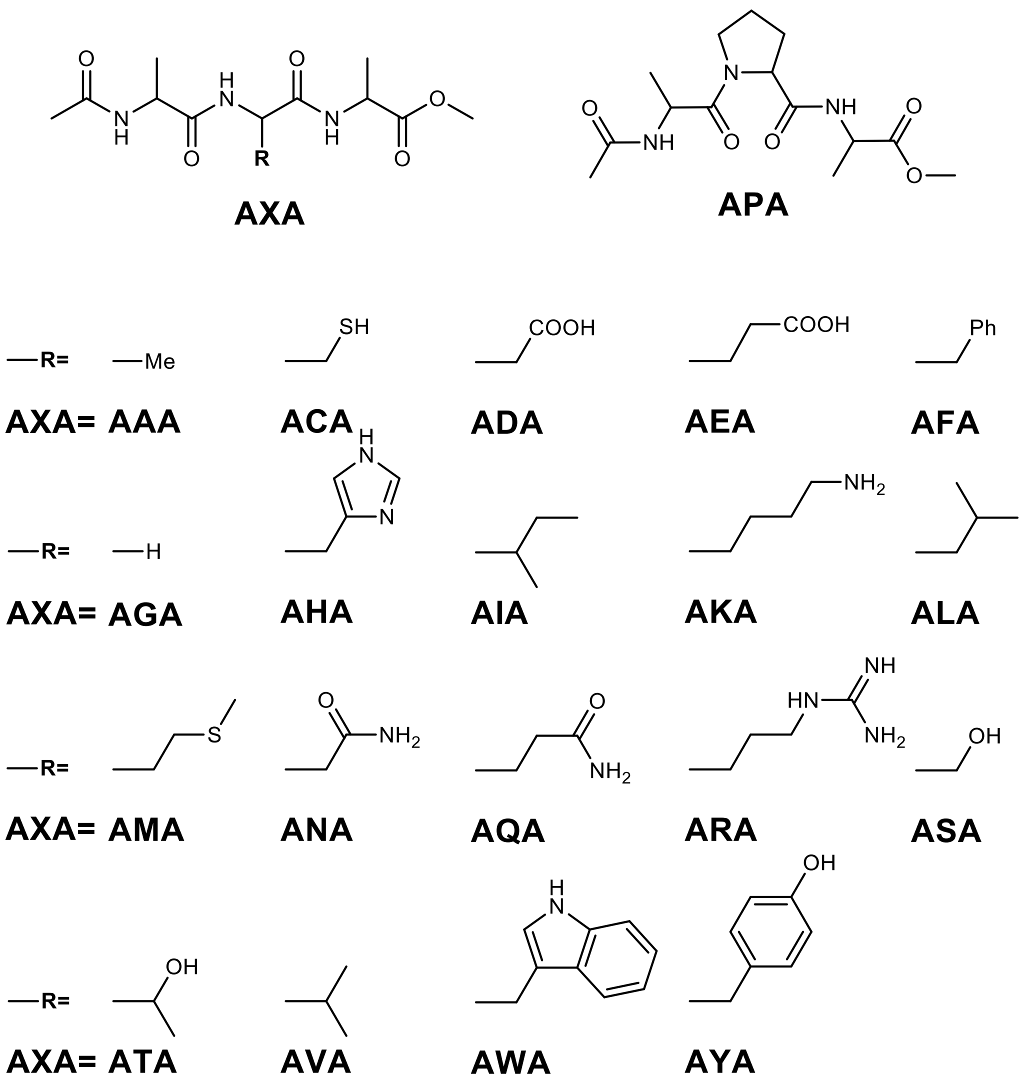

Supplementary Figure 5 | Tripeptides AXA used for assessing photo-reaction kinetics with alkyl diazirine. Here X denotes one of the 20 amino acid residues with various *R* group; the N-terminus of the peptide is acetylated and C-terminus methyl-esterified. Note that though the peptide intends to evaluate the reaction selectivity mimicking a real protein, only enhanced conversion rate may be detected above the intrinsic photo-reaction with AAA.

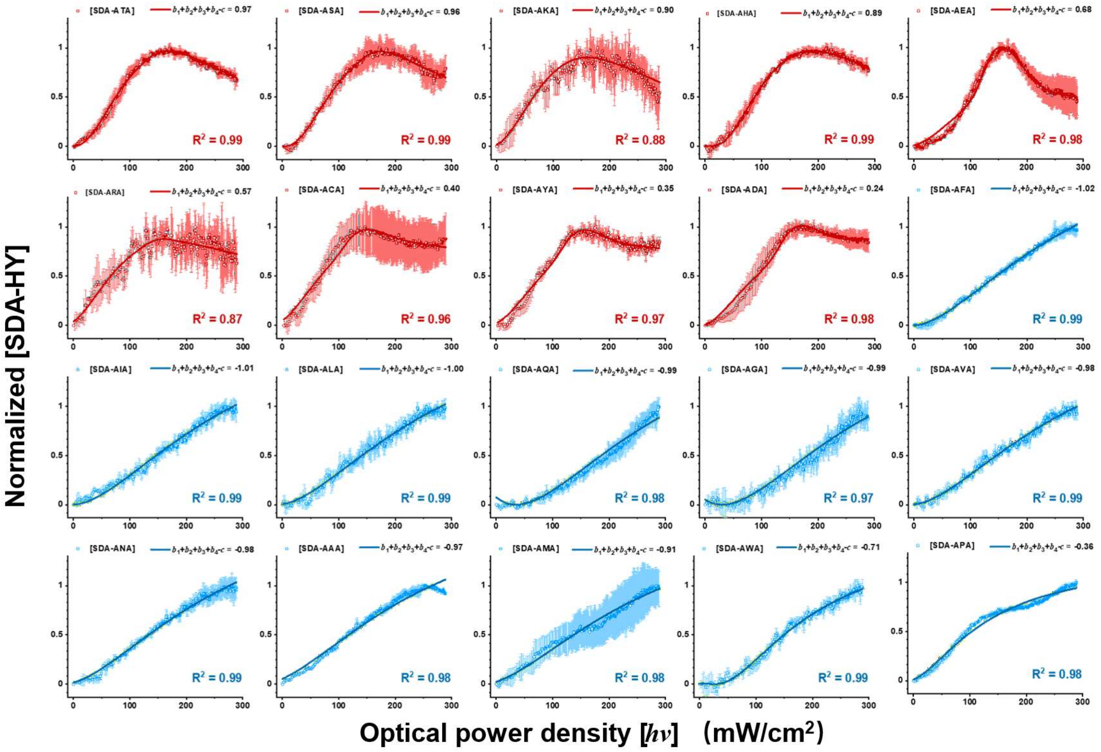

Supplementary Figure 6 | Fitting [SDA-HY] experimental data over optical power density [*hv*]. The experiments were performed for 20 amino acid residues in the terminally blocked tripeptide; each measurement was repeated three times. Polar and non-polar residues are colored red and blue, respectively, as judged by the relative contribution from diazo and carbene intermediate; the fitted values are given in **Supplementary Table 1**. The error bars represent 1 S.D. from experimental measurements for $n = 3$; Pearson correlation coefficients R^2 are given.

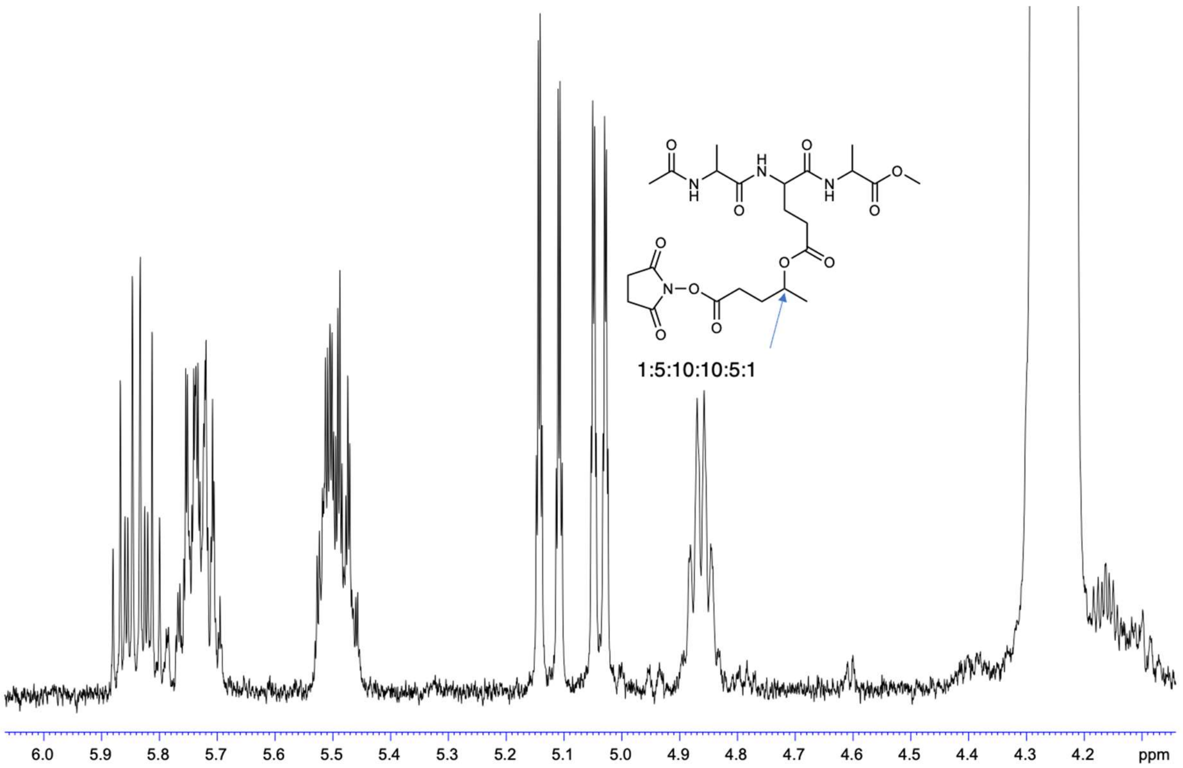

Supplementary Figure 7 | ^{1}H NMR spectrum for the SDA photo-adduct with tripeptide AEA. With the formation of the new ester bond between with the side-chain carboxylate, a new peak appears at ~4.86 ppm (indicated with the blue arrow), with characteristic multiplet for the three-bond scalar couplings with the adjacent methyl and methylene groups.

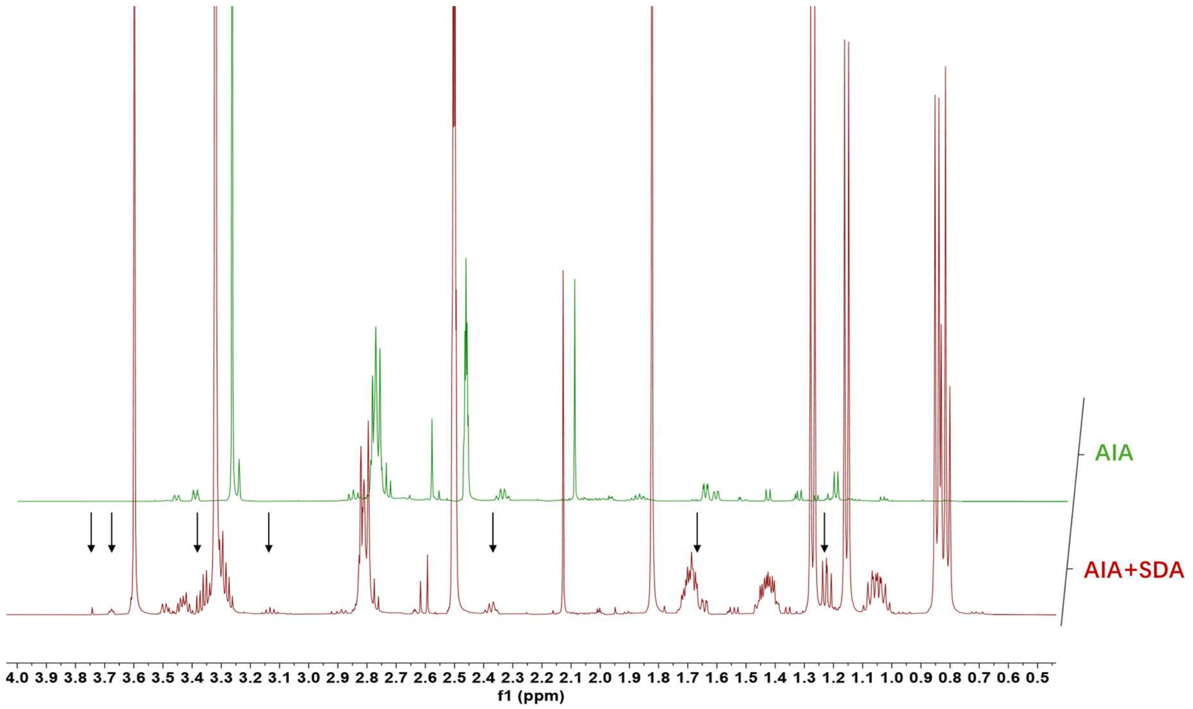

Supplementary Figure 8 | ^{1}H NMR spectrum for the SDA photo-adduct with tripeptide AIA. The arrows indicate new peaks upon photo-reaction.

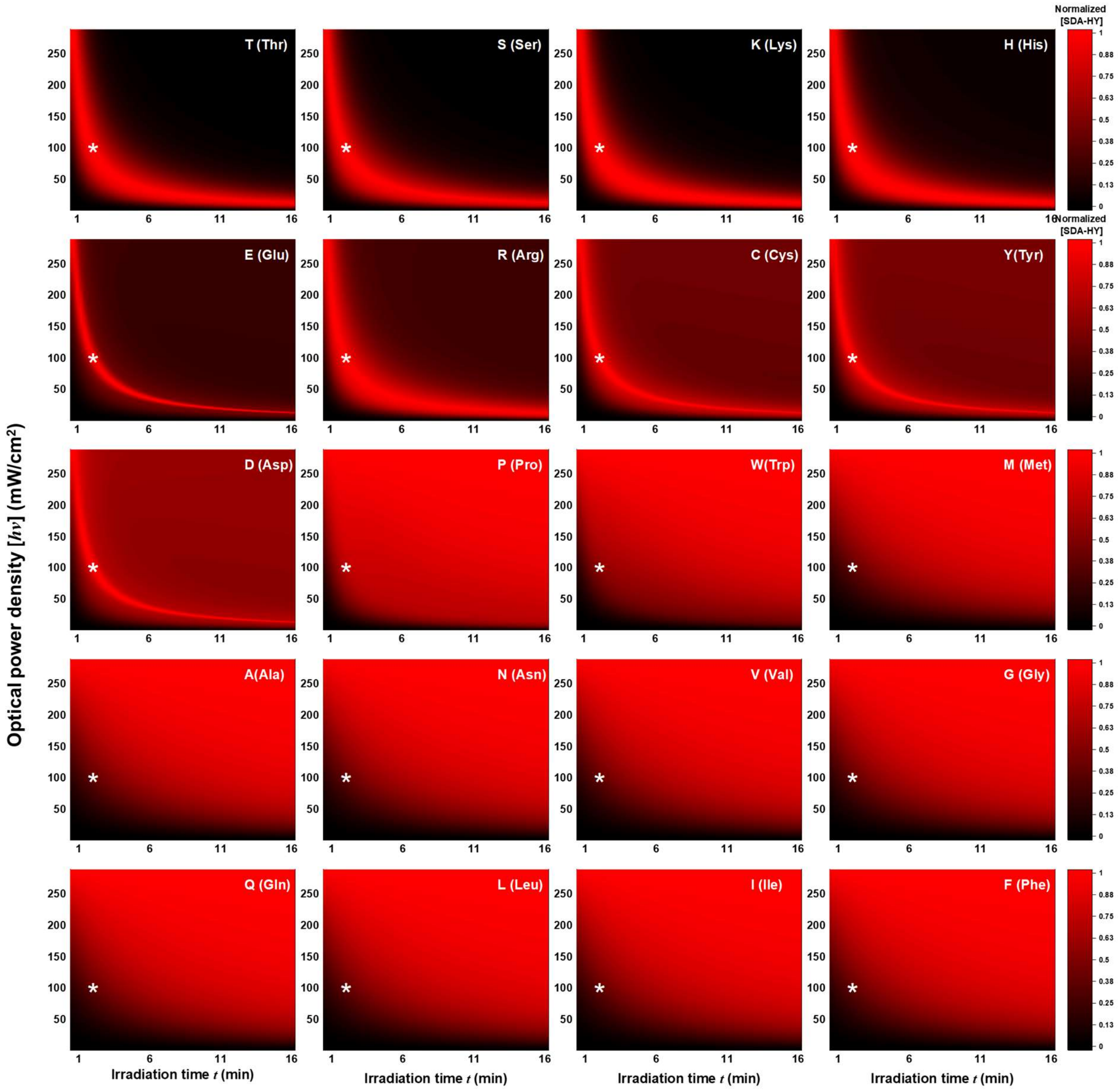

Supplementary Figure 9 | The yield of SDA-HY as a function of irradiation time *t* and optical power density $[h\nu]$. The heat map scale represents the relative yield of the photo-adduct of **SDA-HY**, normalized to the highest value of each residue. The asterisks indicate a preferred value of *t* and $[h\nu]$ for a high yield of **SDA-HY** for polar residues (100 mW/cm^2, 2 min).

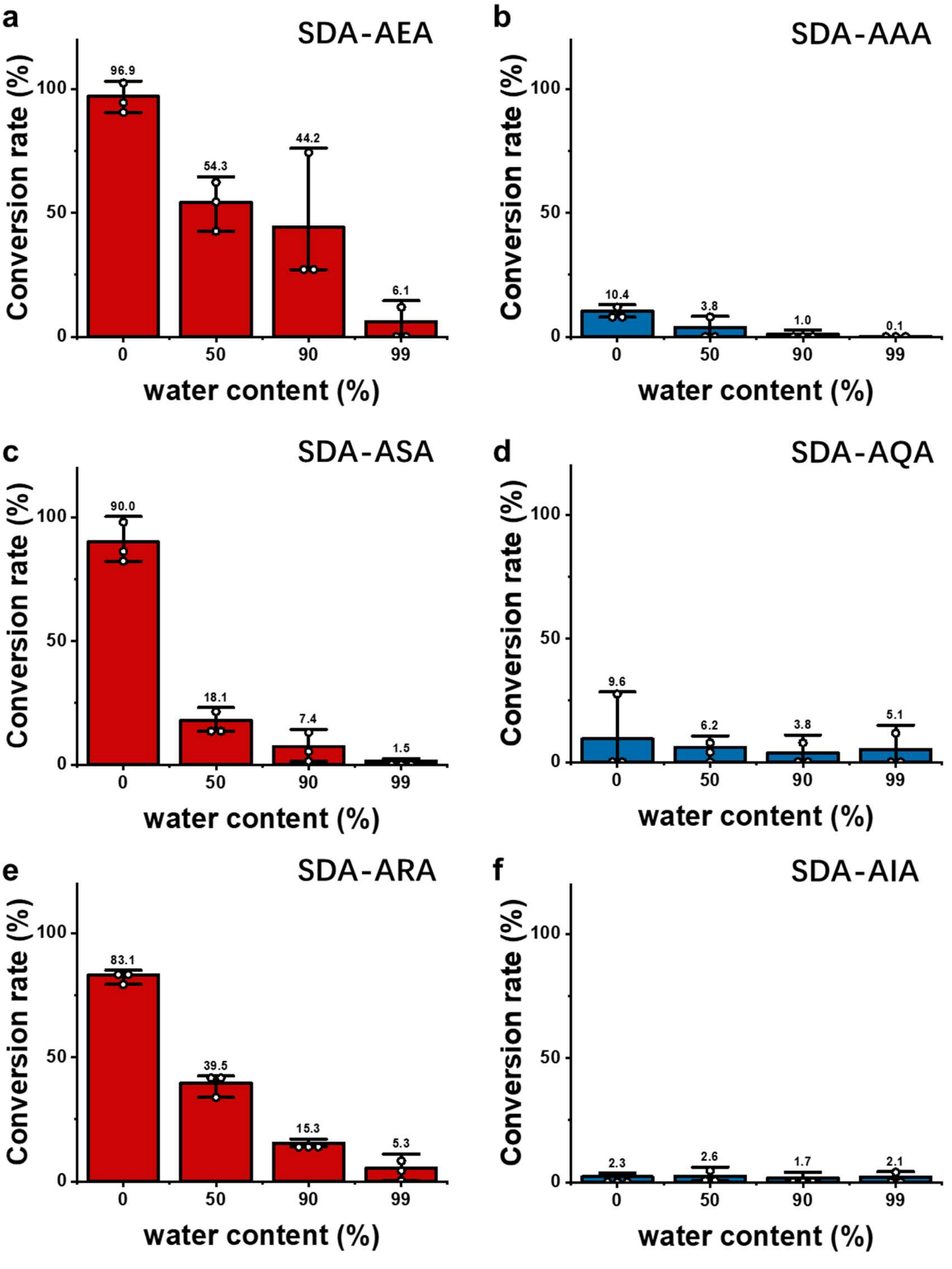

Supplementary Figure 10 | The absolute yield (cf. Fig. 3e) of the SDA photo-adduct decreases with increasing water content (v/v) in the solvent. The conversion rate is computed by the decreasing AXA tripeptide MS signal. Optical power density [$h\nu$] is set at 100 mW/cm^2 with an irradiation time t for 2 minutes. Error bar represent 1 S. D. from triplicated measurements. The hollow circle represents the result of an independent experiment, and their average value is labelled above the bar. Note that the initial yield for **b**, **d** and **f** in water-free solvent (DMSO) is already much lower than **a**, **c** and **e**. Note that AAA tripeptide can also be considered as a background for the diazirine photo-reaction with AEA and other tripeptides. The results suggest that relatively buried protein residues can be better placed to react with diazirine, either with the diazo or carbene-mediated mechanism.

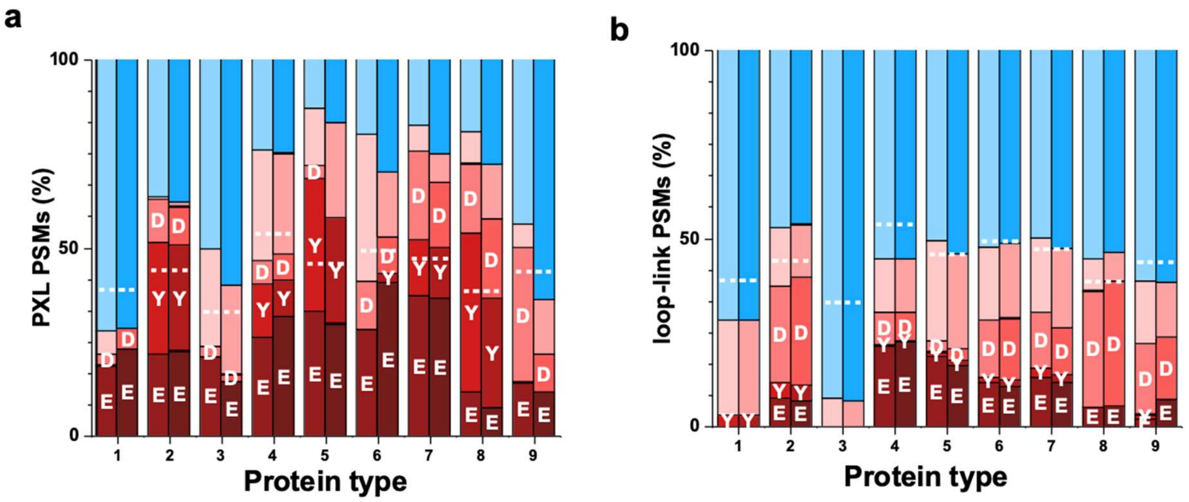

Supplementary Figure 11 | Alkyl diazirine preferentially targets protein polar residues in the test proteins, as evaluated by matched peptide spectra (PSM). **a**, Relative abundance of PSMs for polar residues (red shaded) and non-polar residues (blue shaded), using SDA upon irradiation at an optical power density $[h\nu]$ of 35 mW/cm^2 (left bar, low power) for 10 min or 282 mW/cm^2 (right bar, low power) for 2 min. The dashed line indicates the natural abundance of the polar residues in each protein. D (Asp), E (Glu) and Y (Tyr) are labeled. **b**, Relative abundance of the loop-link PSMs, for non-polar and polar resides in the model proteins upon low- or high-power irradiation. The details of the 9 model proteins are given in Methods.

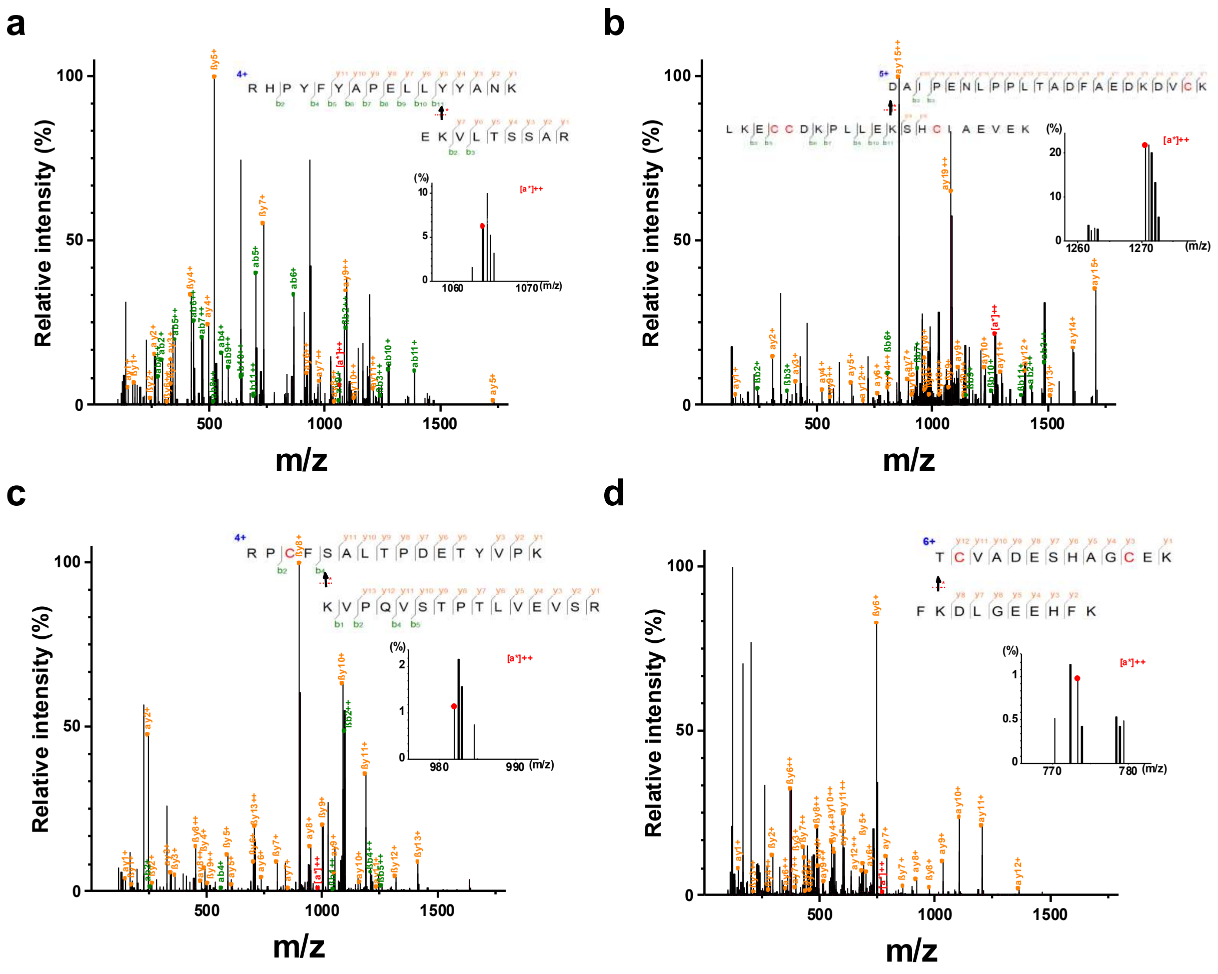

Supplementary Figure 12 | Additional fragmentation of the cross-linked peptide affords the [α] ion. The fragmentation involves the breaking the isopeptide bond to the Lys side-chain, thus enabling further confirmation of the PXLs involving polar residues, namely **a**, Tyr (Y), **b**, Asp (D), **c**, Ser (S), and **d**, Thr (T).

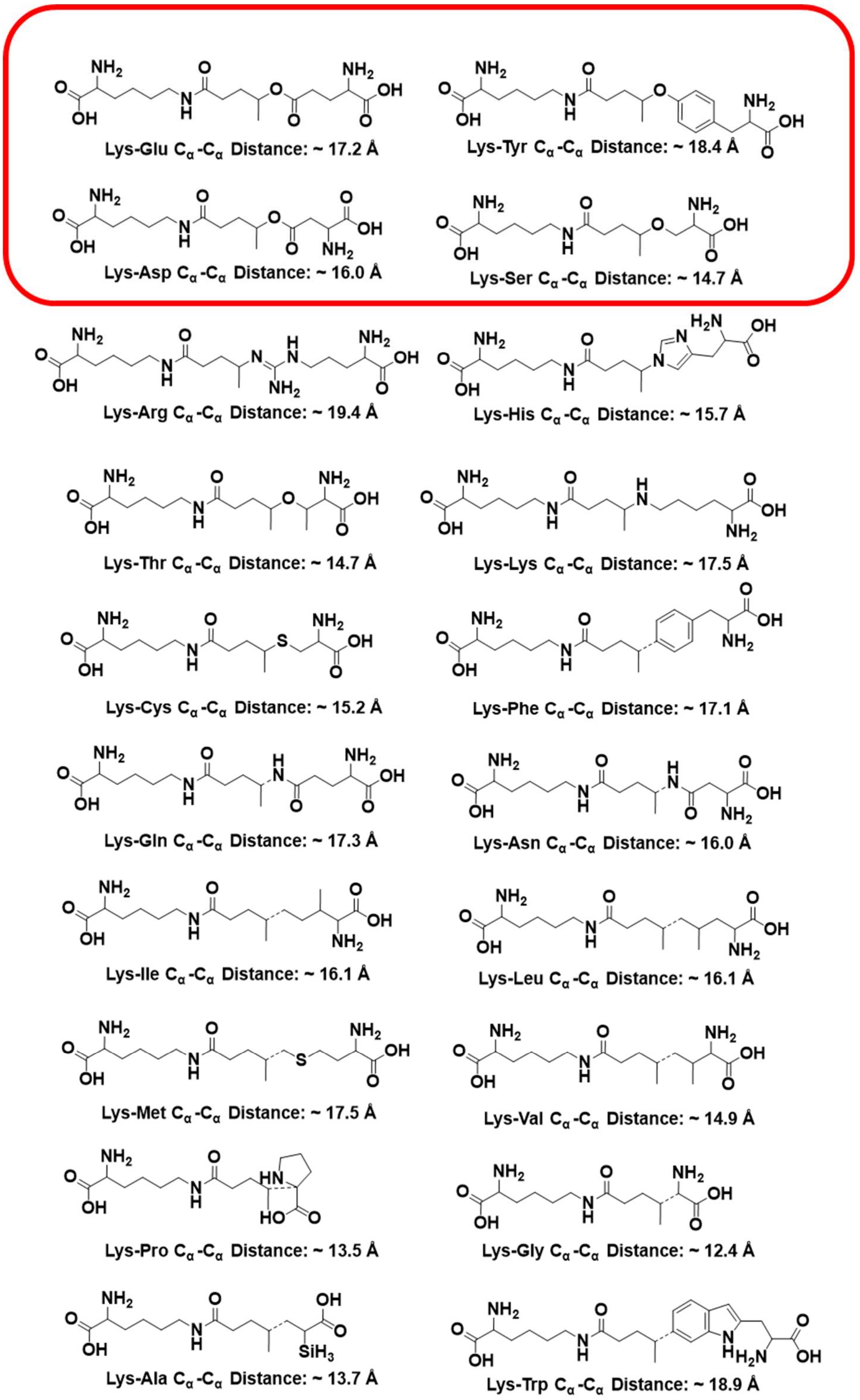

Supplementary Figure 13 | Proposed structures of PXL product involving the side-chain of Lys and another residue. Polar residues largely react with the diazo intermediate, by attacking the tertiary carbon. The four polar residues of which the PXLs can be assigned with highest confidence are boxed. In comparison, the reaction involving non-polar residues yields heterogenous products, with the possible linkages indicated with dashed lines.

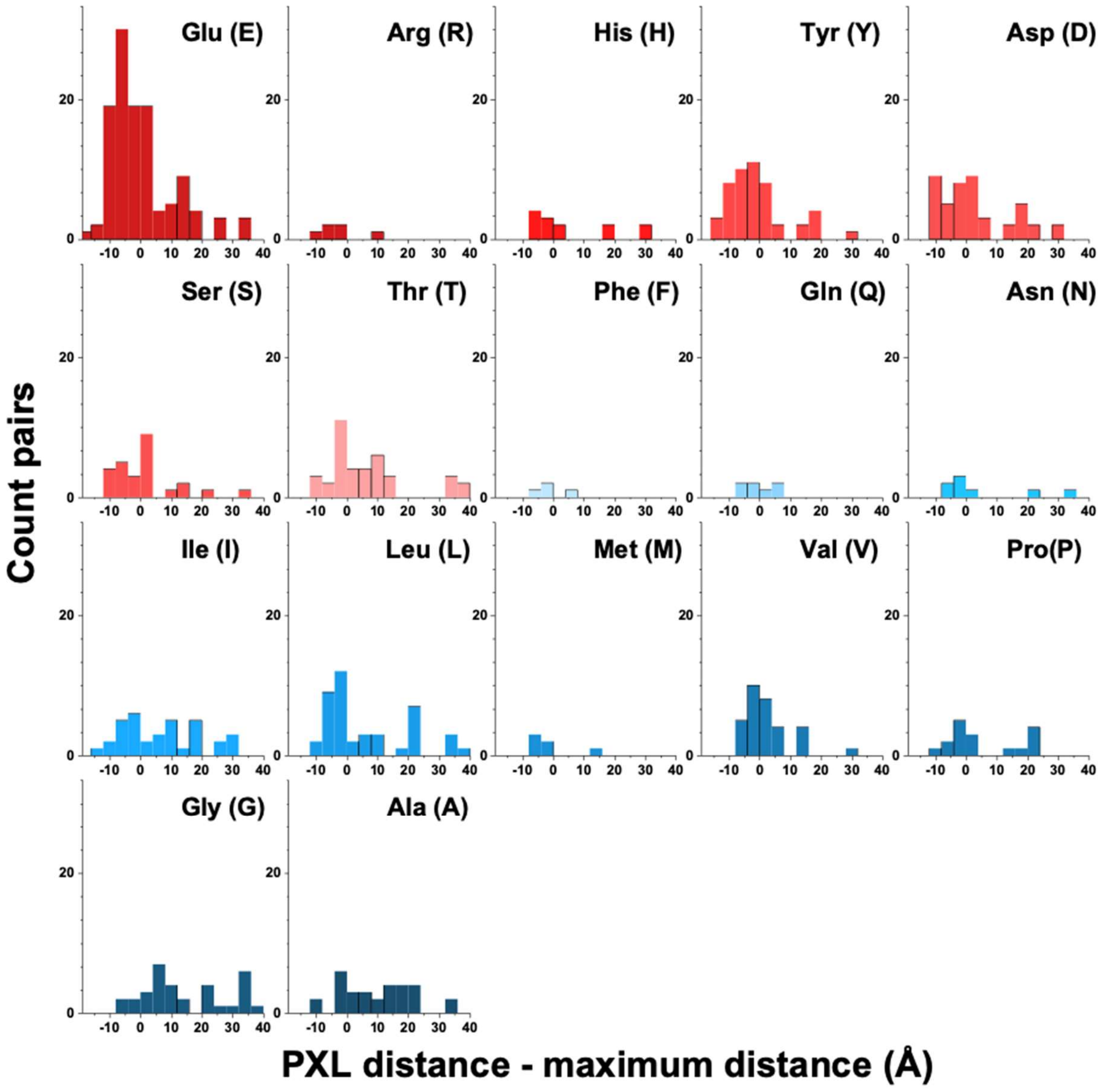

Supplementary Figure 14 | Cα-Cα distance between the PXL residues identified in the model proteins. The distances are computed based on the protein structures, subtracting the maximally allowed theoretical distance (**Supplementary Figure 13**); the relative counts of cross-linked pairs/residues are distributed in 4-Å bins. Note that Lys residues are not included, due to the initial reaction with the NHS moiety, and Cys residues are not included due to alkylation treatment of the cross-linked peptides with 10 mM iodoacetamide. Only few cross-links involving Trp were obtained, which are not included due to lack of statistic meaning. A negative value indicates the consistency between the PXLs and the known protein structure.

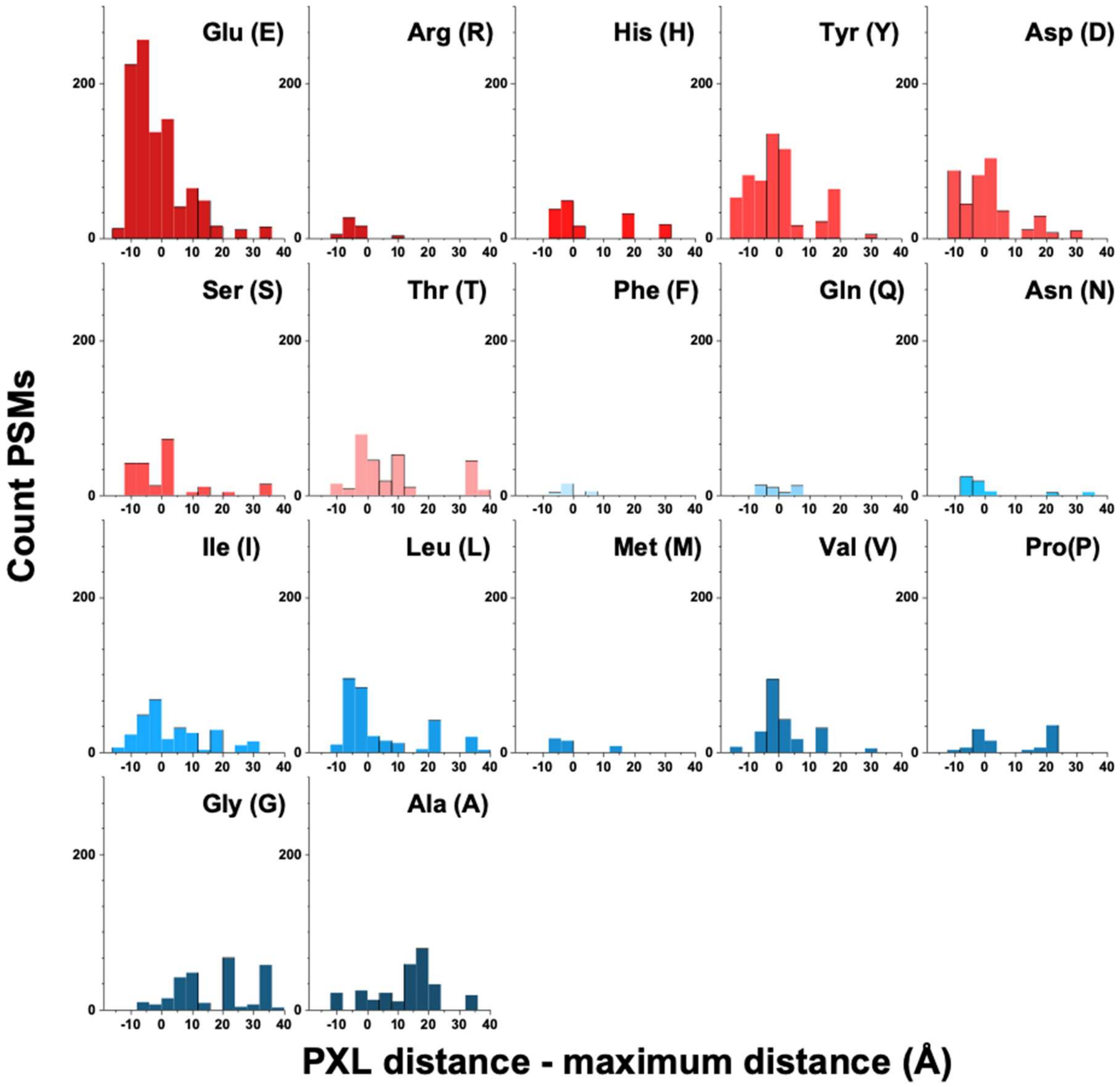

Supplementary Figure 15 | Cα-Cα distance between the PXL residues identified in the model proteins. The distances are computed based on the protein structures, subtracting the maximally allowed theoretical distance (**Supplementary Figure 13**); the relative counts of matched peptide spectra (PSM) are distributed in 4-Å bins. Note that Lys residues are not included, due to the initial reaction with the NHS moiety, and Cys residues are not included due to alkylation treatment of the cross-linked peptides. Few cross-links involving Trp were obtained, which are not included. A negative value indicates the consistency between the PXLs and the known protein structure.

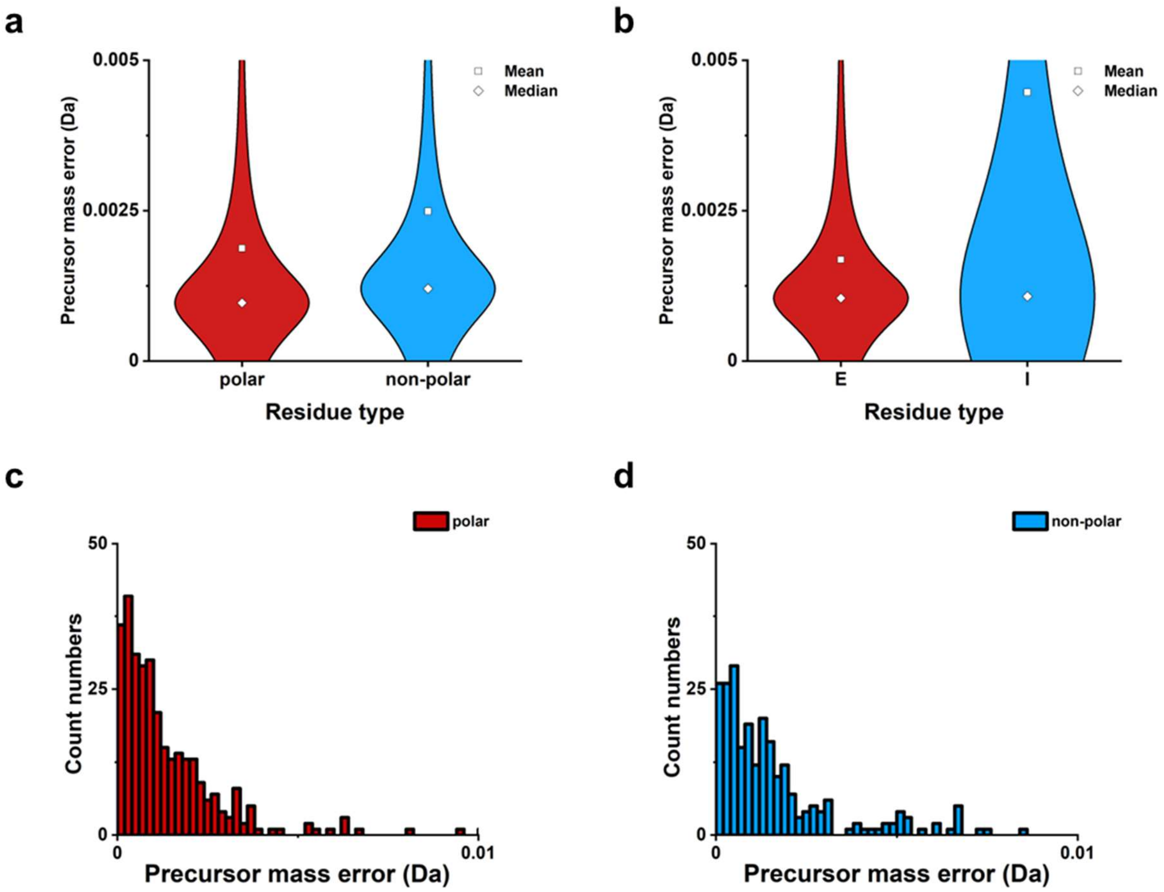

Supplementary Figure 16 | PXLs towards non-polar residues are assigned with higher precursor mass error (PME). In addition, the PME values exhibit larger scattering than those for polar residues. **a**, **b**, PXLs towards non-polar has higher PME in both mean value and median value than polar residues, significant for Ile to Glu. **c**, **d**, PME histogram distribution graph of polar and non-polar residues.

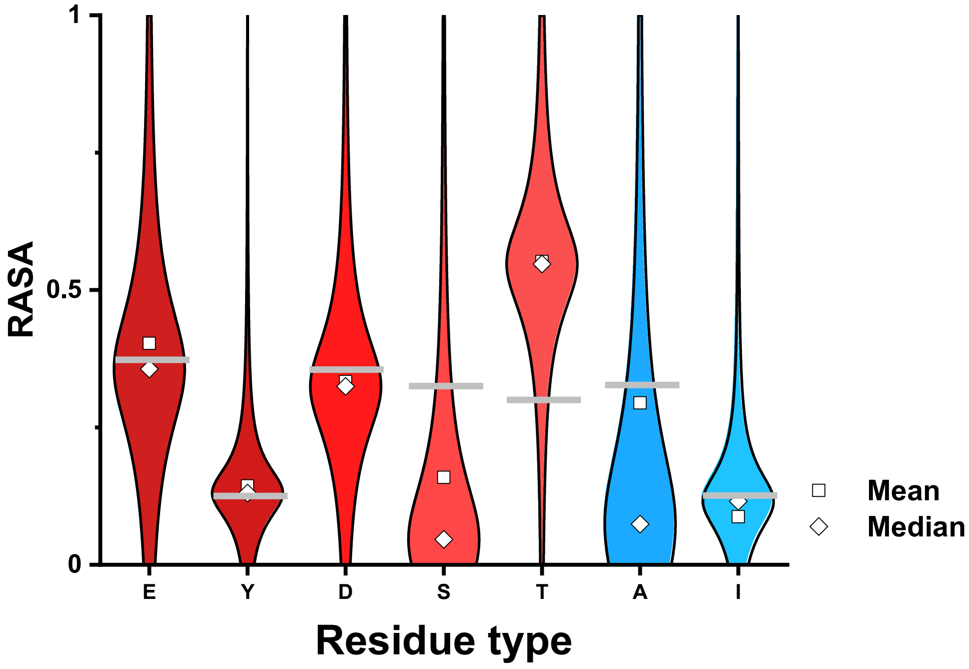

Supplementary Figure 17 | Relative solvent-accessible surface area (RASA) of the residues involved in the PXLs. Except for Thr, most cross-linked polar residues have ASA values below the average values of the model proteins, as indicated by the gray lines. The ASA was computed based on the known structures, which was then divided by the total surface area of each type of residue[1].

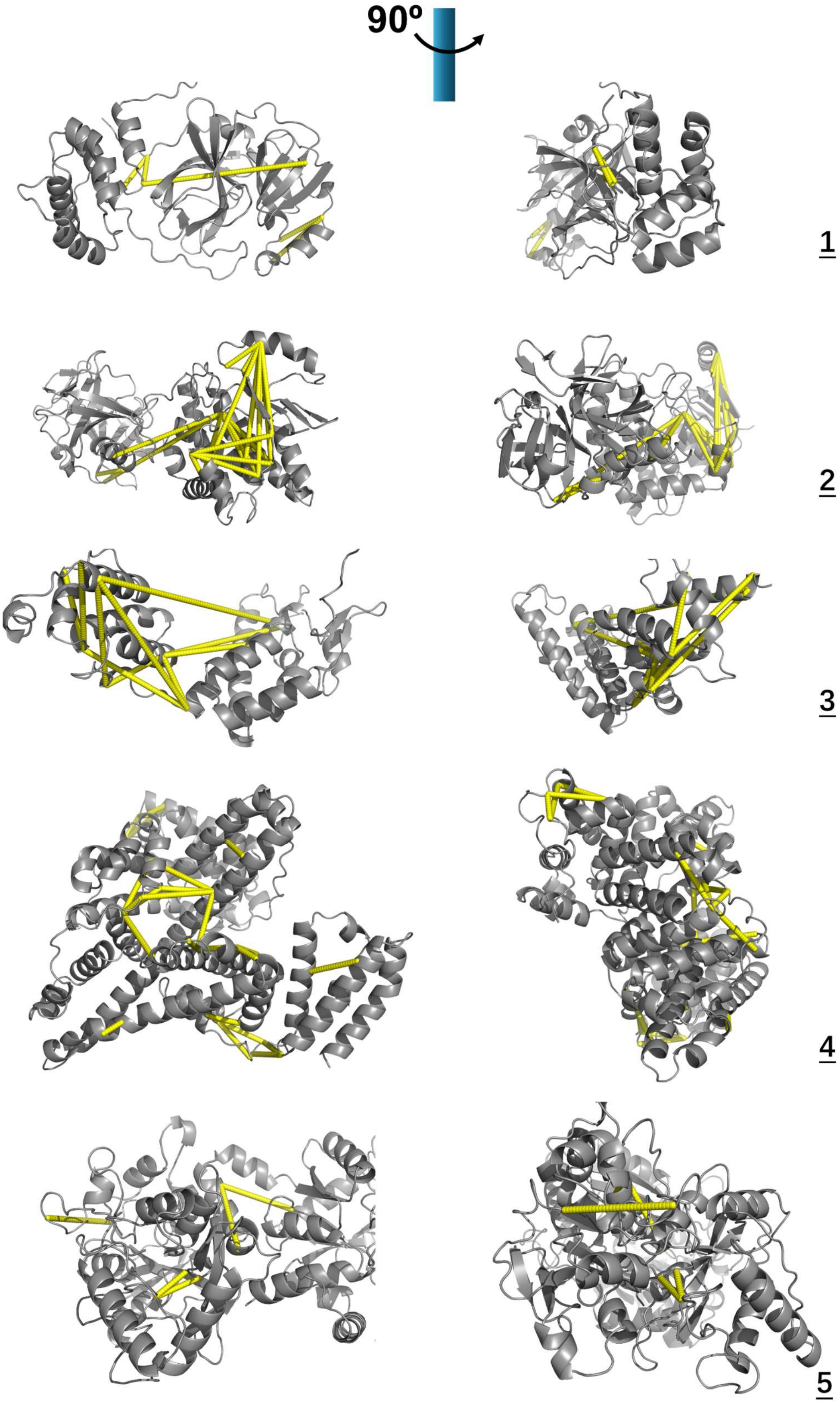
90º
1
2
3
4
5

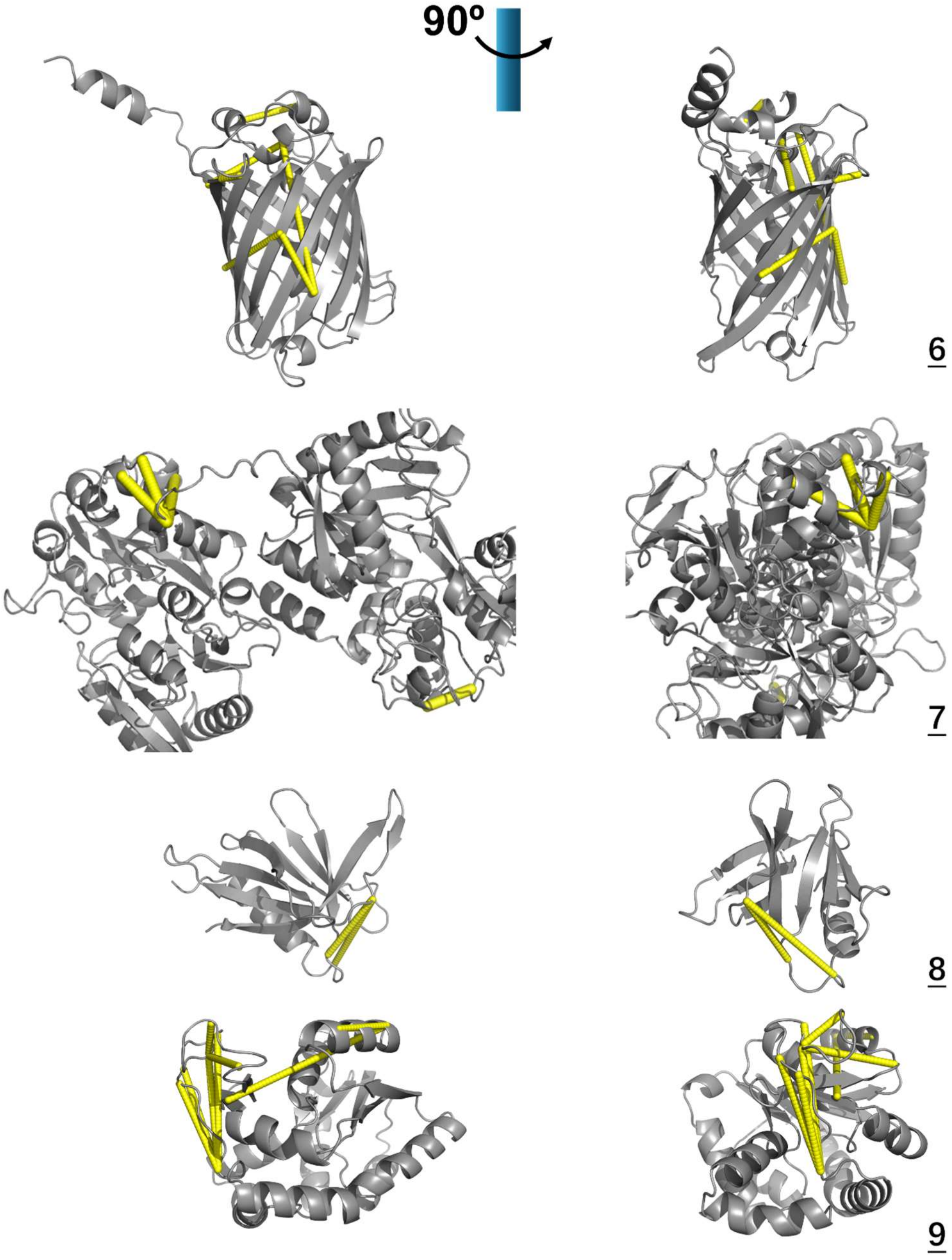

Supplementary Figure 18 | Distance mapping from the PXLs involving polar residues against protein structures. The details of the nine proteins tested are given in Methods. Cross-links are achieved from experiments under optical power density $[h\nu]$ of 35 mW/cm^2 with the irradiation time of 2 min. The PXLs are provided in the Supplementary Data.

Supplementary Tables

Supplementary Table 1 | Relative contribution of the sub-processes to SDA-AXA photo-reaction#

Residue Type (X in AXA)	b_1	b_2	b_3	b_4	c
A	0.00±0.00	0.00±0.00	0.00±0.00	0.00±0.00	0.97±0.01
C	0.00±0.00	0.59±0.02	0.17±0.01	0.00±0.00	0.36±0.01
D	0.46±0.02	0.00±0.00	0.26±0.01	0.00±0.00	0.48±0.02
E	0.00±0.00	0.38±0.01	0.51±0.01	0.00±0.00	0.21±0.01
F	0.00±0.00	0.00±0.00	0.00±0.00	0.00±0.00	1.02±0.00
G	0.00±0.00	0.00±0.00	0.00±0.00	0.00±0.00	0.99±0.01
H	0.13±0.20	0.80±0.17	0.01±0.00	0.00±0.00	0.05±0.05
I	0.00±0.00	0.00±0.00	0.00±0.00	0.00±0.00	1.01±0.01
K	0.11±0.86	0.78±0.75	0.01±0.03	0.00±0.00	0.00±0.20
L	0.00±0.00	0.00±0.00	0.00±0.00	0.00±0.00	1.00±0.01
M	0.00±0.00	0.00±0.00	0.00±0.00	0.00±0.00	0.91±0.00
N	0.00±0.00	0.00±0.00	0.00±0.00	0.00±0.00	0.98±0.00
P	0.31±0.01	0.00±0.00	0.00±0.00	0.00±0.00	0.67±0.01
Q	0.00±0.00	0.00±0.00	0.00±0.00	0.00±0.00	0.99±0.00
R	0.20±0.43	0.52±0.38	0.00±0.00	0.04±0.02	0.19±0.09
S	0.17±0.13	0.72±0.11	0.07±0.01	0.00±0.00	0.00±0.03
T	0.00±0.00	0.92±0.01	0.05±0.01	0.00±0.00	0.00±0.01
V	0.00±0.00	0.00±0.00	0.00±0.00	0.00±0.00	0.98±0.01
W	0.00±0.00	0.18±0.01	0.00±0.00	0.00±0.00	0.89±0.01
Y	0.00±0.00	0.53±0.02	0.21±0.01	0.00±0.00	0.39±0.02

The production of **SDA-AXA** comprises five elementary processes, in which the time t and irradiation power $[h\nu]$ dependent changes of [**SDA-AXA**] can be defined as $b_1 I_{b_1} + b_2 I_{b_2} + b_3 I_{b_3} + b_4 I_{b_4} + c I_c$, in which b_1, b_2, b_3, b_4, and c are weighting factors, though the absolute yield can vary greatly between residues. I_{b_1} and I_{b_2} represent the direct reaction between protonated **AXA** and diazo intermediate and between deprotonated **AXA** and diazo intermediate, respectively, whereas I_{b_3} and I_{b_4} represent the respective proton-catalyzed processes. I_c represents the carbene-mediated reaction.

Supplementary Table 2 | MRM parameters for the in-line analysis of *A* and *D* upon diazirine photolysis

Interface	Nebulizing Gas Flow (L/min)	Heating Gas Flow (L/min)	Interface Temperature (°C)	DL Temperature (°C)	Heat Block Temperature (°C)	Drying Gas Flow (L/min)
ESI	3	10	300	250	400	10

	Precursor m/z	Product m/z	Dwell Time(ms)	Q1 Pre Bias(V)	CE	Q3 Pre Bias(V)
A	304.00	81.00	100.0	20.0	22.0	28.0
D	292.00	81.00	100.0	19.0	23.0	28.0
	236.04	207.10	100.0	24.0	22.0	19.0
	236.05	115.10	100.0	29.0	35.0	20.0

Supplementary Table 3 | MRM parameters for in-line analysis of diazirine photo-reaction with AXA tripeptide

	Precursor m/z	Product m/z	Dwell Time(ms)	Q1 Pre Bias(V)	CE	Q3 Pre Bias(V)
AAA	288.10	175.20	100.0	-21.0	-10.0	-17.0
	288.10	185.20	100.0	-21.0	-10.0	-19.0
	288.10	104.00	100.0	-22.0	-18.0	-20.0
ACA	320.10	207.20	100.0	-14.0	-12.0	-12.0
	320.10	44.10	100.0	-25.0	-38.0	-14.0
	320.10	76.15	100.0	-26.0	-25.0	-29.0
ADA	354.20	251.15	100.0	-10.0	-19.0	-27.0
	354.20	179.20	100.0	-10.0	-23.0	-11.0
	354.20	224.05	100.0	-17.0	-21.0	-23.0
AEA	368.10	262.25	100.0	-15.0	-21.0	-27.0
	368.10	237.20	100.0	-15.0	-24.0	-23.0
AFA	364.20	120.10	100.0	-29.0	-25.0	-20.0
	364.20	251.20	100.0	-30.0	-13.0	-26.0
	364.20	261.25	100.0	-10.0	-11.0	-12.0
AGA	274.10	161.20	100.0	-23.0	-10.0	-29.0
	274.10	104.15	100.0	-11.0	-22.0	-10.0
	274.10	44.10	100.0	-11.0	-39.0	-16.0
AHA	376.10	245.10	100.0	-15.0	-24.0	-25.0
	376.10	273.10	100.0	-15.0	-21.0	-12.0
	376.10	276.15	100.0	-15.0	-25.0	-17.0
AIA	330.10	86.15	100.0	-13.0	-23.0	-15.0
	330.10	227.20	100.0	-14.0	-10.0	-15.0
	330.10	217.20	100.0	-26.0	-11.0	-22.0
AKA	345.20	84.05	100.0	-14.0	-40.0	-16.0
	345.20	129.20	100.0	-29.0	-19.0	-25.0
	345.20	232.25	100.0	-14.0	-17.0	-10.0
ALA	330.10	86.15	100.0	-13.0	-23.0	-14.0
	330.10	227.15	100.0	-14.0	-10.0	-14.0
	330.10	217.25	100.0	-24.0	-12.0	-29.0
AMA	348.10	104.15	100.0	-14.0	-17.0	-10.0
	348.10	245.15	100.0	-14.0	-10.0	-16.0
	348.10	56.20	100.0	-28.0	-32.0	-24.0
ANA	236.05	195.10	100.0	-16.0	-7.0	-17.0

	236.05	138.25	100.0	-16.0	-12.0	-26.0
	236.05	154.10	100.0	-17.0	-11.0	-15.0
APA	314.10	201.20	100.0	-23.0	-12.0	-22.0
	314.10	70.20	100.0	-13.0	-28.0	-12.0
	314.10	44.10	100.0	-13.0	-48.0	-17.0
AQA	367.15	236.15	100.0	-16.0	-24.0	-15.0
	367.15	264.20	100.0	-16.0	-21.0	-17.0
	367.15	150.20	100.0	-16.0	-21.0	-23.0
ARA	373.20	70.20	100.0	-15.0	-39.0	-28.0
	373.20	243.20	100.0	-15.0	-22.0	-25.0
	373.20	114.10	100.0	-15.0	-23.0	-21.0
ASA	304.10	286.10	100.0	-12.0	-10.0	-19.0
	304.10	191.20	100.0	-24.0	-12.0	-19.0
	304.10	60.05	100.0	-12.0	-24.0	-23.0
ATA	318.20	256.30	100.0	-13.0	-22.0	-17.0
	318.20	258.15	100.0	-25.0	-18.0	-25.0
AVA	236.05	195.10	100.0	-16.0	-7.0	-17.0
	236.05	138.25	100.0	-16.0	-12.0	-26.0
	236.05	154.10	100.0	-17.0	-11.0	-15.0
AWA	425.15	294.20	100.0	-11.0	-26.0	-13.0
	425.15	407.15	100.0	-17.0	-25.0	-19.0
	425.15	312.20	100.0	-17.0	-26.0	-21.0
AYA	380.15	136.25	100.0	-16.0	-22.0	-13.0
	380.15	91.20	100.0	-15.0	-54.0	-17.0
	380.15	267.20	100.0	-30.0	-13.0	-18.0

Supplementary Notes

Mathematic rendering for the analysis of diazirine photolysis mechanism

Upon irradiation, the diazirine compound undergoes photolysis reaction through one of the four possible mechanisms as illustrated in **Fig. 1**. Alkyl diazirine ***A*** is converted to the diazo intermediate ***B*** or directly to the carbene intermediate ***C***, with the kinetic rates of $k_1[hv]$ and $k_3[hv]$, respectively. ***B*** absorbs a second photon and transforms to ***C***, with the kinetic rate of $k_2[hv]$. ***C***, a short-lived carbene intermediate[2,3], quickly transforms to an oxidized product ***D*** or other products with the kinetic rate of k_{ox} or k_{others}[4]. The ***C***-to-***D*** conversion ratio *m* can be defined as $k_{ox}/(k_{ox} + k_{others})$.

Model **I** is a simplified version of model **II**, differing for the lack of a direct ***A***-to-***C*** process. Model **III** assumes that k_2 process occurs without irradiation, while model **IV** involving no ***B***-to-***C*** process, which assumption has been held in the previous literature[5,6].

We calculated the kinetic equation of ***A***, ***B*** and ***D***, which can be monitored with MS or NMR. ***A*** becomes depleted upon irradiation following a single-exponential process over *t*, which can be defined as

$$\mathrm{d}[\mathrm{A}]_t = -k_1[hv][\mathrm{A}]_t dt - k_3[hv][\mathrm{A}]_t \mathrm{d}t \quad (1)$$

$$[\mathrm{A}]_t = [\mathrm{A}]_0 exp(-(k_1 + k_3)[hv]t) \ (2)$$

The curves for the concentration of ***B*** and ***D*** include one or two exponential terms (in model **I**, $k_3 = 0$; in model **III**, $k_2{}' = k_2/[hv]$, while $k_2{}' = k_2$ in all other models; in model **IV**, $k_2 = 0$). Thus, [***B***] and [***D***] can be written as

$$\mathrm{d}[\mathrm{B}]_t = k_1[hv][\mathrm{A}]_t \mathrm{dt} - k_2'^{[hv][\mathrm{B}]_t} \mathrm{dt}, with \ [\mathrm{B}]_0 = 0 \ (3)$$

$$[\mathrm{B}]_t = \frac{k_1[\mathrm{A}]_0}{k_1 - k_2{}' + k_3}\left(exp(-k_2{}'[hv]t) - exp(-(k_1 + k_3)[hv]t)\right) \ (4)$$

$$m = k_{ox}/(k_{ox} + k_{othe}\quad) \ (5)$$

$$\mathrm{d}[\mathrm{D}]_t = mk_2'^{[hv]} t\mathrm{d}t + mk_3[hv][\mathrm{A}]_t \mathrm{d}t, with \ [\mathrm{D}]_0 = 0 \ (6)$$

$$[\mathrm{D}]_t = m[\mathrm{A}]_0 \left(1 - \frac{k_1}{k_1 - k_2' + k_3} exp(-k_2'[hv]t) + \frac{k_2' - k_3}{k_1 - k_2' + k_3} exp(-(k_1 + k_3)[hv]t) \right) \quad (7)$$

We can differentiate these models based on MRM data using the power-modulated photo-reaction system, and obtain a best fit for the kinetic parameters. Note that the different appearance of $[\mathrm{D}]_t$ is important to model validation, which varies upon the modulation of optical power density $[hv]$.

The exponent $(k_1 + k_3)[hv]$ can be obtained from the curve of ***A*** with the fitting to the single-exponential function. The curve of ***D*** contains two exponential terms. While it is difficult to fit $k_1[hv]$, $k_2[hv]$ and $k_3[hv]$ directly, $k_2[hv]$ can be fitted from the sectional curve of ***D*** directly, when the value of $1/[hv]$ is smaller than the maximum irradiation time t (with the assumption of $k_2[hv] < (k_1 + k_3)[hv]$), which could be validated with good fitting). With $(k_1 + k_3)[hv]$ and $k_2[hv]$ determined, the whole curve of ***D*** can be fitted to obtain the individual values of $k_1[hv]$ and $k_3[hv]$. As shown in **Fig. 1** and **Fig. 2**, for model **III**, $k_1 + k_3 = 2.32 \times 10^{-4}\ cm^2/(mW \cdot s), k_1 = 9k_3, k_2 = 73 \times 10^{-4}\ s^{-1}$.

Mathematic treatment for diazirine photo-reaction with tripeptide

Multiple elementary reactions can be involved for the generation SDA-HY (protonated form of the tripeptide AXA), as shown in the following Scheme. The sub-processes include I, in which a proton transfers from HY to ***B***; II, III, IV, in which a proton transfers from HY to ***B*** upon the catalysis of a free proton; V, in which ***B*** reacts with free proton; VI, in which elimination reaction leads to the generation of by-products $\boldsymbol{B}_{\mathbf{out}}$.

I.
$$\mathbf{B} + \mathbf{HY} \xrightarrow{k_{\mathrm{I}}} \mathbf{HB^{+}} + \mathbf{Y^{-}} \qquad \text{(slow)}$$

II.
$$\mathbf{HM^{+}} + \mathbf{HY} \underset{k_{\mathrm{II}}^{-1}}{\overset{k_{\mathrm{II}}}{\rightleftharpoons}} \mathbf{HM\text{-}HY^{+}}$$

III.
$$\mathbf{B} + \mathbf{HM\text{-}HY^{+}} \xrightarrow{k_{\mathrm{III}}} \mathbf{HB^{+}} + \mathbf{HM^{+}} + \mathbf{Y^{-}} \qquad \text{(slow)}$$

IV.
$$\mathbf{B} + \mathbf{HM\text{-}HY^{+}} \xrightarrow{k_{\mathrm{IV}}} \mathbf{HB^{+}} + \mathbf{M} + \mathbf{HY}$$

V.
$$\mathbf{B} + \mathbf{HM^{+}} \xrightarrow{k_{\mathrm{V}}} \mathbf{HB^{+}} + \mathbf{M}$$

VI.
$$\mathbf{HB^{+}} + \mathbf{M} \xrightarrow[-N_2]{k_{\mathrm{VI}}} \mathbf{B_{out}} + \mathbf{HM^{+}}$$

VII.
$$\mathbf{HB^{+}} + \mathbf{Y^{-}} \xrightarrow[-N_2]{k_{\mathrm{VII}}} \mathbf{SDA\text{-}HY} \qquad \text{(slow)}$$

VIII.
$$\mathbf{HB^{+}} + \mathbf{HY} \xrightarrow[-N_2]{k_{\mathrm{VIII}}} \mathbf{SDA\text{-}HYH^{+}} \qquad \text{(slow)}$$

IX.
$$\mathbf{M} + \mathbf{SDA\text{-}HYH^{+}} \xrightarrow{k_{\mathrm{IX}}} \mathbf{HM^{+}} + \mathbf{SDA\text{-}HY} \qquad \text{(slow)}$$

Thus, the production of [**SDA-HY**] from diazo intermediate ***B*** and carbene intermediate ***C*** can be written as

$$[\mathrm{SDA-HY}]_{[h\nu],t} = [\mathrm{SDA-HY}]^{\mathrm{B}}_{[h\nu],t} + [\mathrm{SDA-HY}]^{\mathrm{C}}_{[h\nu],t} \quad (8)$$

Here $[\mathrm{SDA-HY}]^{\mathrm{B}}_{[h\nu],t}$ is a complex function related to $[\mathrm{B}]_{[h\nu],t}$, $[\mathrm{HY}]$, and $[\mathrm{H^{+}}]$. For a specific irradiation time *t*, the production rate of [SDA-HY] from ***B*** can be written as

$$\frac{\mathrm{d}[\mathrm{SDA-HY}]^{\mathrm{B}}_{[h\nu],t}}{\mathrm{d}t} = k_{\mathrm{VII}}[\mathrm{HB^{+}}]_{[h\nu],t}[\mathrm{Y^{-}}]_{[h\nu],t} + k_{\mathrm{IX}}[\mathrm{SDA-HYH^{+}}]_{[h\nu],t}[\mathrm{M}] \quad (9)$$

Here $[\mathrm{SDA-HYH^{+}}]_{[h\nu],t}$ is a transient intermediate satisfying the steady-state approximation. For simplicity, with a relatively low photo-reaction yield and with **HY** in excess, [**HY**] can be assumed constant as shown, which leads to

$$0 \equiv \frac{\mathrm{d[SDA-HYH^+]}_{[h\nu],t}}{\mathrm{d}t} = k_{\mathrm{VIII}}[\mathrm{HB^+}]_{[h\nu],t}[\mathrm{HY}] - k_{\mathrm{IX}}[\mathrm{SDA-HYH^+}]_{[h\nu],t}[\mathrm{M}] \quad (10)$$

Equation (10) can then be rearranged as

$$\frac{\mathrm{d[SDA-HY]}^{\mathrm{B}}_{[h\nu],t}}{\mathrm{d}t} = k_{\mathrm{VII}}[\mathrm{HB^+}]_{[h\nu],t}[\mathrm{Y^-}]_{[h\nu],t} + k_{\mathrm{VIII}}[\mathrm{HB^+}]_{[h\nu],t}[\mathrm{HY}] \quad (11)$$

$[\mathrm{HB^+}]_{[h\nu],t}$ is also a short-lived intermediate that satisfies steady-state approximation. Thus, we can obtain the following equation

$$\begin{aligned} 0 &\equiv \frac{\mathrm{d[HB^+]}_{[h\nu],t}}{\mathrm{d}t} \\ &= k_{\mathrm{I}}[\mathrm{B}]_{[h\nu],t}[\mathrm{HY}] + k_{\mathrm{III}}[\mathrm{B}]_{[h\nu],t}[\mathrm{HM-HY^+}]_{[h\nu],t} \\ &+ k_{\mathrm{IV}}[\mathrm{B}]_{[h\nu],t}[\mathrm{HM-HY^+}]_{[h\nu],t} + k_{\mathrm{V}}[\mathrm{B}]_{[h\nu],t}[\mathrm{HM^+}]_{[h\nu],t} - k_{\mathrm{VI}}[\mathrm{HB^+}]_{[h\nu],t}[\mathrm{M}] \\ &- k_{\mathrm{VII}}[\mathrm{HB^+}]_{[h\nu],t}[\mathrm{Y^-}]_{[h\nu],t} - k_{\mathrm{VIII}}[\mathrm{HB^+}]_{[h\nu],t}[\mathrm{HY}] \quad (12) \end{aligned}$$

By omitting the slow processes shown in the Scheme, we can obtain

$$0 \equiv \frac{\mathrm{d[HB^+]}_{[h\nu],t}}{\mathrm{d}t} = k_{\mathrm{IV}}[\mathrm{B}]_{[h\nu],t}[\mathrm{HM-HY^+}]_{[h\nu],t} + k_{\mathrm{V}}[\mathrm{B}]_{[h\nu],t}[\mathrm{HM^+}]_{[h\nu],t} - k_{\mathrm{VI}}[\mathrm{HB^+}]_{[h\nu],t}[\mathrm{M}] \quad (13)$$

Equation (13) can be rearranged to

$$[\mathrm{HB^+}]_{[h\nu],t} = \frac{k_{\mathrm{IV}}[\mathrm{B}]_{[h\nu],t}[\mathrm{HM-HY^+}]_{[h\nu],t} + k_{\mathrm{V}}[\mathrm{B}]_{[h\nu],t}[\mathrm{HM^+}]_{[h\nu],t}}{k_{\mathrm{VI}}[\mathrm{M}]} \quad (14)$$

For the sub-reaction **II** in all possible elementary reactions shown in the Scheme, $[\mathrm{HM-HY^+}]_{[h\nu],t}$ and $[\mathrm{HM^+}]_{[h\nu],t}$ are in rapid equilibrium. Therefore, we can obtain

$$k_{\mathrm{II}}[\mathrm{HM^+}]_{[h\nu],t}[\mathrm{HY}] = k_{\mathrm{II}}^{-1}[\mathrm{HM-HY^+}]_{[h\nu],t} \quad (15)$$

Therefore, $[\mathrm{HB^+}]_{[h\nu],t}$ has a proportional relationship with $[\mathrm{HM^+}]_{[h\nu],t}$ (from (14) and (15)). With charge balance, we can obtain that $[\mathrm{HB^+}]_{[h\nu],t}$ has a proportional relationship with $[\mathrm{Y^-}]_{[h\nu],t}$.

We can then obtain the following equation by combining Equations (11), (14), and (15)

$$\frac{\mathrm{d[SDA-HY]}^{\mathrm{B}}_{[h\nu],t}}{\mathrm{d}t} = k_{\mathrm{VII}}[\mathrm{HB^+}]_{[h\nu],t}[\mathrm{Y^-}]_{[h\nu],t} + k_{\mathrm{VIII}}[\mathrm{HB^+}]_{[h\nu],t}[\mathrm{HY}] = k_{\mathrm{b}}[\mathrm{B}]_{[h\nu],t} \quad (16)$$

As $[\mathrm{HB}^+]_{[h\nu],t}$ has a proportional relationship with $[\mathrm{Y}^-]_{[h\nu],t}$, we can obtain the rate constant k_b, which has the following relationship with $[\mathrm{Y}^-]$ (m_1 and m_2 are scaling factors):

$$k_\mathrm{b} \propto m_1[\mathrm{Y}^-]_{[h\nu],t} + m_2[\mathrm{Y}^-]^2_{[h\nu],t} \ (17)$$

If $k_\mathrm{I} \gg k_\mathrm{III}$ (t' is an integration variable):

$$\frac{\mathrm{d}[\mathrm{Y}^-]_{[h\nu],t}}{\mathrm{d}t} = k_\mathrm{I}[\mathrm{B}]_{[h\nu],t}[\mathrm{HY}] \ (18)$$

$$[\mathrm{Y}^-]^\mathrm{I}_{[h\nu],t} \propto \int_0^t [\mathrm{B}]_{[h\nu],t'}\,\mathrm{d}t' (19)$$

If $k_\mathrm{I} \ll k_\mathrm{III}$:

$$\frac{\mathrm{d}[\mathrm{Y}^-]_{[h\nu],t}}{\mathrm{d}t} = k_\mathrm{III}[\mathrm{B}]_{[h\nu],t}[\mathrm{HM}-\mathrm{HY}^+]_{[h\nu],t} \propto [\mathrm{B}]_{[h\nu],t}[\mathrm{Y}^-]_{[h\nu],t} \ (20)$$

$$[\mathrm{Y}^-]^\mathrm{III}_{[h\nu],t} \propto \exp\left(\int_0^t [\mathrm{B}]_{[h\nu],t'}\,\mathrm{d}t'\right) \ (21)$$

We can thus obtain the following simplified equations, with m_1, m_2, n_1, and n_2 the pre-factors

$$[\mathrm{Y}^-]_{[h\nu],t} = n_1[\mathrm{Y}^-]^\mathrm{I}_{[h\nu],t} + n_2[\mathrm{Y}^-]^\mathrm{III}_{[h\nu],t} \ (22)$$

$$I_{b_1} = \int_0^t m_1\, n_1[\mathrm{B}]_{[h\nu],t'}[\mathrm{Y}^-]^I_{[h\nu],t'}dt' \ (23)$$

$$I_{b_2} = \int_0^t m_2\, n_1[\mathrm{B}]_{[h\nu],t'}\left([\mathrm{Y}^-]^I_{[h\nu],t'}\right)^2 dt' \ (24)$$

$$I_{b_3} = \int_0^t m_1\, n_2[\mathrm{B}]_{[h\nu],t'}[\mathrm{Y}^-]^{III}_{[h\nu],t'}dt' \ (25)$$

$$I_{b_4} = \int_0^t m_2\, n_2[\mathrm{B}]_{[h\nu],t'}\left([\mathrm{Y}^-]^{III}_{[h\nu],t'}\right)^2 dt' \ (26)$$

Thus, the diazo-mediated production of **SDA-HY** can be written as

$$[\mathrm{SDA-HY}]^{\mathrm{B}}_{[h\nu],t} = \int_0^t k_b[\mathrm{B}]_{[h\nu],t'}\, dt' \approx b_1 I_{b_1} + b_2 I_{b_2} + b_3 I_{b_3} + b_4 I_{b_4} \quad (27)$$

With $[\mathrm{B}]_{[h\nu],t} = \frac{k_1[A]_0}{k_1-k_2}\left(exp(-k_2[h\nu]t) - exp(-(k_1)[h\nu]t)\right)$, the following equations can be obtained for I_{b_1} and I_{b_2}

$$I_{b_1} = \frac{A_0^2\begin{pmatrix} e^{-2[h\nu](k_1+k_2)t_1}\left(k_1\left(-e^{[h\nu]k_1t_1}\right) + (k_1-k_2)e^{[h\nu](k_1+k_2)t_1} + k_2e^{[h\nu]k_2t_1}\right)^2 + \\ 2[h\nu]k_1k_2\left(e^{-[h\nu]\nu k_2t_1} - e^{-[h\nu]k_1t_1}\right) \\ \begin{pmatrix} -\frac{k_1\left(e^{-[h\nu]k_2t_1} - e^{-[h\nu]k_2t_2}\right)}{[h\nu]k_2} + \frac{k_2\left(e^{-[h\nu]k_1t_1} - e^{-[h\nu]k_1t_2}\right)}{[h\nu]k_1} \\ +k_1(-t_1) + k_1t_2 + k_2t_1 - k_2t_2 \end{pmatrix} \end{pmatrix}}{2[h\nu]^2(k_1-k_2)^2k_2^2} \quad (28)$$

$$I_{b_2} = \frac{A_0^2\begin{pmatrix} e^{-2[h\nu](k_1+k_2)t_1}\left(k_1\left(-e^{[h\nu]k_1t_1}\right) + (k_1-k_2)e^{[h\nu](k_1+k_2)t_1} + k_2e^{[h\nu]k_2t_1}\right)^2 + \\ 2[h\nu]k_1k_2\left(e^{-[h\nu]k_2t_1} - e^{-[h\nu]k_1t_1}\right) \\ \begin{pmatrix} -\frac{k_1\left(e^{-[h\nu]k_2t_1} - e^{-[h\nu]k_2t_2}\right)}{[h\nu]k_2} + \frac{k_2\left(e^{-[h\nu]k_1t_1} - e^{-[h\nu]k_1t_2}\right)}{[h\nu]k_1} \\ +k_1(-t_1) + k_1t_2 + k_2t_1 - k_2t_2 \end{pmatrix} \end{pmatrix}}{2[h\nu]^2(k_1-k_2)^2k_2^2} \quad (29)$$

Here, $(t_2 - t_1)$ represents the time for the sample flowing in the PFA tube to MS detection. The parameters can be obtained from experimental observables by using the in-line power-modulated photo-reaction system with these parameters: $k_1 = 0.000232\ \mathrm{cm}^2/(\mathrm{mW}\cdot\mathrm{s})$, $k_2 = 0.000073\ \mathrm{cm}^2/(\mathrm{mW}\cdot\mathrm{s})$, $t_1 = 59$ s, $t_2 = 80$ s, and $A_0 = 1$.

Though I_{b_3} and I_{b_4} lack analytical mathematical solutions, their numerical results can be fitted, resulting the following equations:

$$I_{b_3} \text{ or } I_{b_4} = A \times \left(1/\left(1 + exp(-(x - x_c + w_1/2)/w_2)\right)\right) \times \left(1 - 1/\left(1 + exp(-(x - x_c - w_1/2)/w_3)\right)\right) \quad (30)$$

For I_{b_3}, $x_c = 170.82$, $A = 1.288$, $w_1 = 63.0$, $w_2 = 11.93$, $w_3 = 20.20$. For I_{b_4}, $x_c = 170.60$, $A = 1.286$, $w_1 = 44.7$, $w_2 = 9.17$, $w_3 = 13.32$. Excellent fitting could be obtained （$R^2 = 0.9999$）.

For the production of **SDA-HY** from the carbene intermediate ***C***, the time-dependent change of [**SDA-HY**] can be written as:

$$[\mathrm{SDA-HY}]^{\mathrm{C}}_{[hv],t} = [A]_0\left(1 - \frac{k_1}{k_1 - k_2}exp(-k_2[hv]t) + \frac{k_2}{k_1 - k_2}exp(-k_1[hv]t)\right) \times \frac{k_c}{k_c + k_{ox} + k_{others}} = cI_c \quad (31)$$

In brief, according to the k_1, k_2 given by Model I (a simplified model to Model II) and experiments, we can deduce and calculate the normalized yield of the product [**SDA-HY**] – [*hv*] curve of ***B*** or ***C*** at *t* = 1 min. Assuming that the **HY** is in excess, its concentration can be regarded as a constant; whereas the concentration of ***B*** and the cumulative yield of ***C*** are the functions of k_1, k_2, [*hv*], and *t*. The diazo-mediated reaction can be dissected into elementary reactions involving the transfer of proton, which is thus related to the capability of **HY** to provide proton (b_1 and b_2 processes) and to the proton concentration in the solvent (the enhancement of b_3 over b_1, and the enhancement of b_4 over b_2). Thus, the acidity of the protein side-chain (pKa value) and the buffer (pH) can have an effect on the kinetic rate of diazo-mediated photo-reaction.

Supplementary References

S 1 Tien, M. Z., Meyer, A. G., Sydykova, D. K., Spielman, S. J. & Wilke, C. O. Maximum allowed solvent accessibilites of residues in proteins. *PLoS ONE* **8**, e80635(2013).

S2 Admasu, A. *et al.* A laser flash photolysis study of p-tolyl(trifluoromethyl)carbene. *J. Chem. Soc., Perkin Trans. 2*, 1093-1100(1998).

S3 Toscano, J. P., Platz, M. S. & Nikolaev, V. Lifetimes of simple ketocarbenes. *J. Am. Chem. Soc.* **117**, 4712-4713(1995).

S4 O'Brien, J. G. K., Jemas, A., Asare-Okai, P. N., Am Ende, C. W. & Fox, J. M. Probing the mechanism of photoaffinity labeling by dialkyldiazirines through bioorthogonal capture of diazoalkanes. *Org. Lett.* **22**, 9415-9420(2020).

S5 Iacobucci, C. *et al.* Carboxyl-photo-reactive ms-cleavable cross-linkers: Unveiling a hidden aspect of diazirine-based reagents. *Anal. Chem.* **90**, 2805-2809(2018).

S6 Piersimoni, L., Kastritis, P. L., Arlt, C. & Sinz, A. Cross-linking mass spectrometry for investigating protein conformations and protein–protein interactions—a method for all seasons. *Chem. Rev.* **122**, 7500-7531(2021).
